# Regioselective Sequential Modification of Chitosan via Azide-Alkyne Click Reaction: Synthesis, Characterization, and Antimicrobial Activity of Chitosan Derivatives and Nanoparticles

**DOI:** 10.1371/journal.pone.0123084

**Published:** 2015-04-30

**Authors:** Atif Sarwar, Haliza Katas, Siti Noradila Samsudin, Noraziah Mohamad Zin

**Affiliations:** 1 Centre for Drug Delivery Research, Faculty of Pharmacy, Universiti Kebangsaan Malaysia, Kuala Lumpur Campus, Jalan Raja Muda Abdul Aziz, Kuala Lumpur, Malaysia; 2 Novel Antibiotic Research Group, Faculty of Health Sciences, Universiti Kebangsaan Malaysia, Kuala Lumpur Campus, Jalan Raja Muda Abdul Aziz, Kuala Lumpur, Malaysia; Duke University Marine Laboratory, UNITED STATES

## Abstract

Recently, the attention of researchers has been drawn toward the synthesis of chitosan derivatives and their nanoparticles with enhanced antimicrobial activities. In this study, chitosan derivatives with different azides and alkyne groups were synthesized using click chemistry, and these were further transformed into nanoparticles by using the ionotropic gelation method. A series of chitosan derivatives was successfully synthesized by regioselective modification of chitosan via an azide-alkyne click reaction. The amino moieties of chitosan were protected during derivatization by pthaloylation and subsequently unblocked at the end to restore their functionality. Nanoparticles of synthesized derivatives were fabricated by ionic gelation to form complexes of polyanionic penta-sodium tripolyphosphate (TPP) and cationic chitosan derivatives. Particle size analysis showed that nanoparticle size ranged from 181.03 ± 12.73 nm to 236.50 ± 14.32 nm and had narrow polydispersity index and positive surface charge. The derivatives and corresponding nanoparticles were evaluated *in vitro* for antibacterial and antifungal activities against three gram-positive and gram-negative bacteria and three fungal strains, respectively. The minimum inhibitory concentration (MIC) of all derivatives ranged from 31.3 to 250 µg/mL for bacteria and 188 to1500 µg/mL for fungi and was lower than that of native chitosan. The nanoparticles with MIC ranging from 1.56 to 25 µg/mLfor bacteria and 94 to 750 µg/mL for fungi exhibited higher activity than the chitosan derivatives. *Chitosan O-(1-methylbenzene) triazolyl carbamate *and *chitosan O-(1-methyl phenyl sulfide) triazolyl carbamate* were the most active against the tested bacterial and fungal strains. The hemolytic assay on erythrocytes and cell viability test on two different cell lines (Chinese hamster lung fibroblast cells V79 and Human hepatic cell line WRL68) demonstrated the safety; suggesting that these derivatives could be used in future medical applications. Chitosan derivatives with triazole functionality, synthesized by Huisgen 1,3-dipolar cycloaddition, and their nanoparticles showed significant enhancement in antibacterial and antifungal activities in comparison to those associated with native, non-altered chitosan.

## Introduction

Recently, there has been a remarkable surge in research to explore properties and pharmaceutical applications of chitosan, a positively charged polymer. Chitosan is known to possess numerous biological properties such as antibacterial [1,2], antifungal [3], anticancer [4], anticholesterolemic [5], wound healing properties [6], biocompatibility, and biodegradability [7]. In addition, chitosan has also been utilized as a mucoadhesive in the promotion of transmucosal absorption [8], a bioadhesive agent in nasal drug delivery and for other mucosal routes [9,10], as well as a delivery system for plasmid DNA (pDNA) [11], and genetic material [12].

Chemically modified derivatives of chitosan have received increasing interest over the past few years due to their improved chemical, biological, and functional properties over unmodified chitosan. These include better solubility in organic and aqueous solutions over a wide range of pH, improved biocompatibility, better complexation properties with pDNA or siRNA, enhanced antimicrobial activity, and reactivity with other substances[13].

Introduction of small functional groups such as alkyl or carboxymethyl [13] on the chitosan structure has been shown to significantly increase the solubility of chitosan at neutral and alkaline pH without altering the cationic nature of chitosan. The free amino and hydroxyl groups on the C-3 and C-6 carbon units are the two reactive groups amenable for modification in chitosan. It is known that a bulky *N*-phthaloyl group at the C-2 position of chitosan contributes to selective reaction at the C-6 primary hydroxyl group due to steric hindrance as previously reported by others [14,15]. It is interesting to note that most of the previous chitosan modifications had been carried out on primary amino groups, and these could potentially affect the antimicrobial, amine-dependent bioadhesion and pH-dependent properties of chitosan. In addition, the cationic properties of chitosan are attributed to the amino group, and solubilization of chitosan occurs through the protonation of this functional group. To bypass these issues, our approach was to selectively modify the secondary alcohol group on chitosan by a well-defined organic reaction known as Huisgen 1,3-dipolar cycloaddition, one of the most useful click reaction or Sharpless click reaction[16], thereby conserving the amino groups.

Click chemistry is one of the recent approaches aimed at accelerating the synthesis of drug-like molecules [17]. Reactions between molecules, designed using click chemistry are faster, require minimal or no purification, exhibit high tolerance of functional groups, are regiospecific, and produce quantitative yields. Insensitivity of the reaction to the solvents and simple reaction conditions are additional advantages of this technique in comparison to the previously used modifications to obtain chitosan derivatives such as sulfation [18,19], phosphorylation, carboxymethylation, acylation [18,20], Schiff`s base formation and alkylation [18,21]. Given these substantial benefits of click chemistry, it is now a widely used practice in chemistry research, including polymer chemistry, material sciences, and pharmaceutical sciences. In addition, triazole derivatization has been extensively studied for its biological activities such as antibacterial, antifungal, anticancer, and antimycobacterial properties [22,23].

The present work focuses on the non complex derivatization of chiotsan and their corresponding nanoparticles that are predicted to have increased antibacterial and antifungal activities. A selective activity against bacterial and fungal cells coupled with a lack of toxicity towards eukaryotic cells position these novel chitosan derivatives as prospective candidates for further biomedical applications.

## Materials and Methods

### Chemicals and reagents

Low molecular weight (LMW) chitosan powder, 85% deacetylated was purchased from Sigma-Aldrich (USA). Penta-sodium tripolyphosphate (TPP), Muller-Hinton (MH) broth, and agar were supplied by Merck (Germany). Acetic acid, sodium hydroxide, phthalic anhydride, dimethyl formamide (DMF), tetrahydrofuran (THF), 1,1′-carbonyldiimidazole (CDI), proparglyamine, copper (II) acetate, sodium ascorbate, 1-azidoadamantane, azidobenzene solution, 1-azidomethyl-2-methyl benzene solution, azidomethyl phenyl sulphide, 2-azidomethyl-1-Boc-pyrrolidine, ethylenediaminetetraacetic acid (EDTA), hydrazine monohydrate were also supplied by Sigma-Aldrich (USA). All chemicals and reagents were of highest analytical grade and used directly without further purification.

### Synthesis of Chitosan derivatives

#### N-Phthaloylation of chitosan

**Fig 1:**
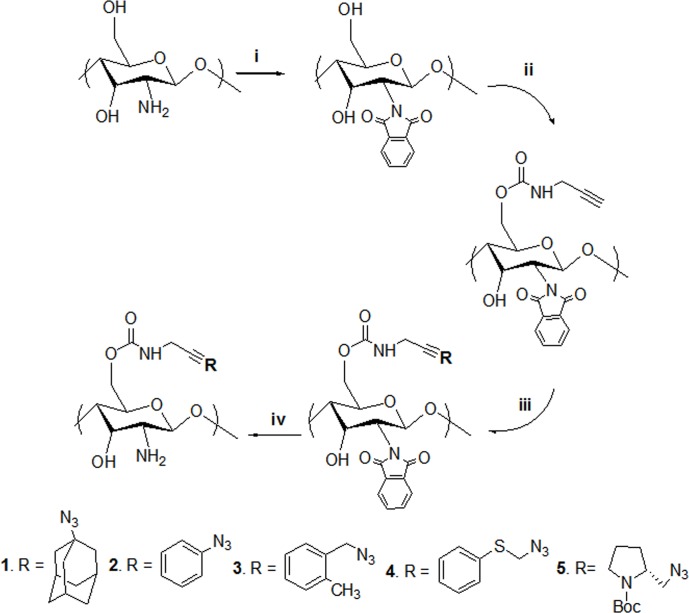
Synthetic scheme of chitosan derivatives. Synthetic scheme of chitosan azide functional derivatives.(i) phthalic anhydride, DMF 5%(v/v), 8h, N_2_ atmosphere,120°C, (ii) THF,CDI, 5h, N_2_ atmosphere, 40°C, propargylamine, THF,24h, 25°C, (iii) sodium ascorbate, copper (II) acetate, (1-azidoadamantane / azidobenzene/ 1-azidomethyl-2-methyl benzene / azidomethyl phenyl sulphide / 2-azidomethyl-1-boc pyrolidine), tertbutanol/ water, 24h, 25°C, (iv) NH_2_NH_2_.H_2_O,water, 18h, 100°C.

*N*-phthaloyl-chitosan was synthesized using a previously reported method [24]. The synthesis procedure of derivatives has been shown in (Fig 1). Chitosan (5.0 g, 31 mmol) and phthalic anhydride (13.8 g, 93.18 mmol) were suspended in 125 mL of DMF containing 5%v/v water and the mixture was heated to 120°C under agitation on a magnetic stirrer and nitrogen atmosphere for 8 h. The reaction mixture was then cooled to room temperature before adding it to ice water. The resultant light brown precipitate was collected by filtration, washed with cold methanol, and air dried for 24 h. The product yield was ~7.0g.

#### Alkynation of N-phthaloyl-chitosan

In the second step, *N*-phthaloyl-chitosan O-prop-2-ynyl carbamate was prepared as described [25]. *N*-phthaloyl-chitosan (3.0 g, 10 mmol) was suspended in 45 mL CDI solution (30 mg/ml in THF) and the mixture was subjected to gentle stirring at 40°C under nitrogen atmosphere for 5 h. At the end of reaction, resultant precipitate was collected by filter funnel and thoroughly washed with THF to remove the excess CDI and transferred to a new flask containing 30 mL propargylamine solution (30 mg/mL in THF) and stirred at room temperature for 24 h. Following this, the solid fraction was filtered, washed with cold methanol, and air dried. Then the resultant product *N*-phthaloyl-chitosan O-prop-2-ynyl carbamate was reacted with azide compounds.

#### Cu[1] catalyzed azide-alkyne cycloaddition of N3 groups

*N*-phthaloyl-chitosan O-prop-2-ynyl carbamate (2.0 g, 4.5 mmol), sodium ascorbate (0.60 g, 3.2 mmol), and copper acetate (0.30 g, 2.25 mmol) were suspended in 80 mL of (1:1, v/v) *tert*-butanol/H_2_O. To this, (4.5 mmol) each of the following azides was added (for individual derivative): 1-azidoadamantane (0.8g), azidobenzene (0.54g), 1-azidomethyl-2-methyl benzene (0.66g), azidomethyl phenyl sulfide (0.74g), 2-azidomethyl-1-Boc-pyrrolidine (0.1g); and the reactions were carried out for 12 h at room temperature under constant agitation.

Solid fractions comprising the products chitosan *O*-(adamantane) triazolyl carbamate, chitosan *O*-(benzene) triazolyl carbamate, chitosan *O*-(1-methylbenzene) triazolyl carbamate, chitosan *O*-(1-methyl phenyl sulfide) triazolyl carbamate, chitosan *O*-((*R*) (1-methyl)-1-Boc-pyrrolidine) triazolyl carbamate were collected by filtration, rigorously washed with 0.1 M EDTA first, followed by methanol wash, and air-dried.

#### Removal of N-Phthaloyl protection

The *N*-phthaloyl protective group was removed by subjecting each resulting product (0.75 g) to hydrazine monohydrate (5 mL, 90 mmol in 50 mL ethanol), at 40°C under continuous stirring for 24 h. The subsequent derivatives were collected on a filter funnel, washed twice with ethanol and water, and finally lyophilized to obtain dry powder.

### Instrumental characterization of chitosan derivatives

#### FT-IR Spectroscopy

Infrared spectra were recorded at room temperature using an FT-IR spectrometer (Spectrum Perkin-Elmer Japan Co. Ltd., Tokyo). All the samples were recorded as derivative-potassium bromide (KBr) pellets prepared by blending 2 mg of previously dried (overnight at 60°C) powdered polymer and 200 mg of previously dried (at 105°C for 24 h) KBr. Spectral values were obtained by accumulation of 16 scans for wavelength range 400–4000 cm^-1^ with resolution of 4 cm^-1^.

#### NMR spectroscopy

^1^H NMR spectra were recorded at room temperature by using DMSO-d_6_ and deuterium oxide (D_2_O) as solvents (Bruker AVANCE 500 spectrometer). Chemical shifts were reported as part per million (ppm).

#### Elemental analysis (EA)

The C and N content of the chitosan derivatives was ascertained using an elemental analyzer (ElementarVario EL III, Hanau, Germany), and the obtained data was used to calculate the DS.

#### TGA

The thermal stability of samples was evaluated using a thermal analyzer (Perkin-Elmer STA-6000, Waltham, USA). For analysis, 8 mg of each sample was analyzed at a temperature range of 50 to 650°C at a heating rate of 10°C /min under a continuous nitrogen flow (20 mL/min).

#### DSC

A diamond differential scanning calorimeter (Perkin-Elmer Waltham USA) was used to obtain DSC data. For each sample approximately 5 mg of lyophilized powder was sealed and analyzed at a temperature range from 0 to 300°Cunder nitrogen flow capacity of 20 mL/min at a heating rate of 10°C/min.

#### SEM

The chitosan and synthesized freeze dried chitosan derivatives were analyzed by SEM. The samples were coated by using a gold sputter coater under an argon atmosphere and mounted onto the aluminum stub of a LEO 1450 VP scanning electron microscope. All the images were recorded at 50 μm and 2000x magnification.

### NP generation and physical characterization

Chitosan derivatives were formulated into NP by ionic cross linking using TPP in a previously described method [26] with some modifications. Accordingly, 0.8 mL of TPP solution (0.1% w/v in distilled water) was added to 4.0 mL of 0.2% solution of chitosan derivatives [derivatives 1, 2 and 3 dissolved in acetic acid (1% v/v) while derivatives 4 and 5 in distilled water] and subject to agitation (using magnetic stirrer at 900 rpm) at room temperature for 30 min. Subsequently, the spontaneously formed NPs were separated by ultracentrifugation at 35,000×*g* at 10°Cfor 30 min and the pellets were re-suspended. Morphology of the nanoparticles was viewed under a TEM (FEI Tecnai, Biotwin, Netherland). Measurement of mean particle size (Z-average), zeta potential (ζ-potential), and PDI was performed by Zetasizer Nano ZS-90 (Malvern, UK). The measurements were made in triplicates at room temperature.

#### Morphology of treated bacteria

Cultures of *E*. *coli* and *S*. *aureus* were centrifuged, washed, and suspended in 0.1 M sodium phosphate buffer (pH-7.4) to obtain an absorbance of 0.4 at 600 nm. The prepared nanoparticles were mixed with bacterial suspensions at a ratio of 1:1 (v/v) and incubated for 2 h at 37°C followed by centrifugation at 10,000x*g* for 10 min at 4°C. The resulting pellets were fixed with 3.0% (v/v) glutaraldehyde in 0.1 M PBS (pH-7.4) followed by two washes with 0.1 M PBS (pH-7.4). The pellets were post-fixed with 1% w/v OsO_4_ in 0.1 M PBS (pH- 7.4) for 1 h followed by three washes with PBS as used previously. Pellets were then dehydrated individually in a graded series of ethanol solutions (30, 50, 70, 80, 90, and 100%, v/v) for 10 min at 4°C prior to embedding in Agar 100 Resin. Thin sections of the specimens were prepared with a diamond knife on Ultracut (Ultramicrotome LE1CA UC6, Austria) and double stained with lead citrate and saturated uranyl acetate. Finally, the grids were examined under a TEM (FEI Tecnai, Biotwin) at an operating voltage of 120 kV[27].

### Biological evaluation of derivatives and NPs

#### Determination of antibacterial activity

Antibacterial activity of chitosan derivatives and their NPs was assessed against three strains each of gram positive (*Staphylococcus aureus*, *Bacillus subtilis*, and *Bacillus cereus*)and gram-negative bacteria (*Escherichia coli*, *Pseudomonas aeruginosa*, and *Acinetobacter schindleri*). The MIC was determined by the microtiter broth dilution method [28]. Briefly, 100 μL of bacterial inocula prepared by adjusting an overnight culture to ~10^5^ cells/mL in Muller Hinton Broth (MHB) were mixed with 100 μL of serial two-fold dilutions of each sample in MH broth in a 96-well plate to give a final concentrations of 1000 μg/mL, and incubated at 37°C for 24 h. Antibacterial activity was determined on the basis of turbidity, which is considered as an indicator of bacterial growth. Following incubation, individual results were assessed visually; presence of turbidity indicated no activity against microorganisms. The MIC was defined as the lowest concentration at which no visible cell growth was observed. For determination of minimum bactericidal concentration (MBC), a 10-μL sample from the wells with no visible growth was spread on MH agar plates. MBC was stated as the concentration with maximum dilution displaying no growth on agar plates after 48 h incubation at 37°C. Two independent experiments were performed to ensure reproducibility of results [22].

#### Determination of antifungal activity

Antifungal properties of derivatives and NPs were tested using a modified microdilution method [21] against *Aspergillus niger*, *Fusarium solani*,and *Candida albicans* [29],and compared to the positive control, amphotericin B. Potato dextrose agar and potato agar broth were used as fungal growth medium. Fungal spores were washed off from agar plates with sterile 0.85% saline solution containing 0.1% (v/v) Tween 80 and adjusted to a concentration of ~10^5^ cells /mL. 100 μL of sample solution was pipetted into 96-well micro titer plate with a final concentration of 3000 μg/mL followed by serial dilution. One hundred microliters of fungal suspensions were inoculated into each well and plates were incubated for 72 h, at 37°C for *C*. *albicans* and at 28°C for *A*. *niger* and *F*. *solani*. Following incubation, each well was examined for visible growth and MIC was assessed as the lowest concentration of sample at which there was no visible growth in the wells. To determine minimum fungicidal concentration (MFC), 10μL of samples from the wells of the MIC plates were spread onto the Petri dishes containing potato dextrose agar. The dishes were incubated for 72 h at optimal growth temperature for each fungal strain (provided previously). MFC was determined as the concentration of sample at which no visible fungal growth was observed [22,30].

#### Cell viability of V-79 and WRL-68

Chinese hamster lung fibroblast cell line V79 (ATCC, USA) and Human hepatic cell line WRL68 were cultured separately in Dulbecco’s Modified Eagle Medium (DMEM) supplemented with 10% fetal bovine serum (FBS) (Sigma Aldrich, USA) and 1% penicillin-streptomycin(ThermoFisher Scientific, USA) at 37°C with 5% carbon dioxide in a humidified-atmosphere. Cell density was maintained at 5 × 10^4^ cells per well in a 96-well plate. After overnight incubation, cells were exposed to chitosan derivatives for 24 h. The initial concentrations of the inoculated samples were 3 mg/ml, which were serially diluted up to four times to observe a range of concentrations at which the cytotoxicity effects of each derivative could be monitored. Following exposure, media was replaced after washing the cells twice with PBS. AlamarBlue reagent (10 μL) was added into the wells and following 48 h of incubation, absorbance at 570 nm was determined using a microplate reader (NanoQuant infinite M200 PRO, Tecan, Switzerland) to assess the amount of dye incorporated in living cells. The percent of cell viability was determined using the following formula[31]:

$$\%\;Cell\;Viability\;=\;\left(\frac{A_{570}\;of\;cells\;with\;samples}{A_{570}of\;control}\right)\times 100$$

#### Hemolytic Assay

Hemolytic activity of synthesized chitosan derivatives on mouse erythrocytes (RBCs) was measured by a modified method [32,33]. Fresh blood samples were immediately collected in heparin containing tubes to prevent coagulation. Maximum hemolysis was determined by adding 1% Triton X-100 (positive control) to a sample containing cells while PBS pH-7.4 was used as negative control. The positive control would show total lysis of erythrocytes (~ 100%), whereas the negative control would have very minimal lysis of erythrocytes (to determine the degree of spontaneous lysis). The erythrocytes were harvested by centrifugation at 2000 rpm for 10 min at 4°C and washed three times in PBS pH-7.4 to obtain a pure suspension of erythrocytes. Erythrocytes were finally resuspended in PBS to produce 1% suspension. 100 μL of various concentrations of chitosan derivatives were added to 96-well microplates containing 100 μL of erythrocytes suspension in triplicate. The derivatives mixed with erythrocytes were incubated at 37°C for 1 hour in a water bath and then centrifuged at 2000 rpm for 10 min at 4°C. Supernatants were transferred to 96-well microplates and the absorbance at 450 nm was determined by using a spectrophotometric microplate reader (NanoQuant infinite M200 PRO, Tecan, Switzerland) to measure the extent of red blood cell lysis. The hemolysis percentage HC_50_ was calculated by the following equation:

$${HC}_{50}\left(\%\right)\;=\;\frac{\left(Abs-{Abs}_{0}\right)}{\left({Abs}_{100}-{Abs}_{0}\right)}\;\times 100$$

where, Abs, Abs_0_ and ‘Abs_100_ represented absorbance of sample, negative control and positive control, respectively.

### Statistical analysis

All studies were conducted in triplicate and data were expressed as mean±standard deviation. Data from different experimental groups were compared by a One-way analysis (ANOVA) using SPSS 16. Results with p<0.05 were considered statistically significant.

## Results and Discussion

### Chemical modification of chitosan

Chitosan is a naturally found polymer that has a wide application owing to its abundant biodegradability. The pharmaceutical use and pharmacological activities of chitosan have been exploited by modifying its basic structure.

Numerous chitosan derivatives have been synthesized by modification of its primary amino groups as they tend to be more reactive than the hydroxyl groups [25]. However, this reactivity of the amino group is one of the special properties that increase its utility in pharmaceutical sciences. Protonation of the primary amino group facilitates solubility of chitosan in acidic conditions. Moreover, the reactive amino group is crucial in maintaining the biological functionality of chitosan. In our attempt to introduce controlled modifications in the hydroxyl group of chitosan, the first step was to protect the reactive amino moiety by adding a phthaloyl group on it. This *N*-phthaloylation process is important for subsequent chemoselectiveC-6 modification and *N*-phthaloylation was confirmed by the Fourier transform infrared (FTIR) band indicating the presence of phthalimide group. Moreover, the solubility of *N*-phthaloyl chitosan in various organic solvents such as dimethyl sulfoxide (DMSO), *N*-methyl-2-pyrrolidone (NMP), and dimethylformamide (DMF) facilitated subsequent modifications, while allowing for terminal unblocking of these amino groups by hydrazine monohydrate [24].

Click chemistry is a relatively newer, better, and controlled approach for chitosan modification. The copper-catalyzed azide-alkyne cycloaddition (CuAAC) is a simple procedure that is easy to perform and provides quantitative yields of final products, which require minimal purification [34].

Prior to coupling with an alkyne group (proparglyamine), the hydroxyl group in N-phthaloyl chitosan has to be activated by a coupling reagent. In order to achieve this, we used carbonyldiimidaziole (CDI), a chemical extensively used for coupling reactions in the modifications of different polysaccharides such as dextran, cellulose, and chitosan [35].

Proparglyamine was selected as the alkyne conjugate because of its stability and hydrophilicity. Five commercially available small chain azide compounds were introduced and coupled with the alkyne group with the help of copper (II)-ascorbate system to promote the click reaction. The cycloaddition process was carried out overnight under gentle agitation at 25°C to minimize the risk of depolymerization of chitosan backbone, which may be attributed to the use of copper (II)-ascorbate system. EDTA was used to wash precipitates in order to remove the excess copper after the cycloaddition process. The azide-alkyne click-reaction completed with the synthesis of triazolyl derivatives. All the synthesized chitosan azoloyl derivatives exhibited an acceptable solubility in organic and organic-aqueous solutions. However, in weak acidic medium, their solubility was lower in comparison to native chitosan, and this can be attributed to decreased hydrophilicity due to the addition of aromatic groups.

Derivatives 4 and 5 were soluble in distilled water, and this feature coupled with preservation of the cationic nature of chitosan by introduction of the azole ring could play a significant role in their antimicrobial actions. In previously reported alkyne-azide click modifications of chitosan, the azo donor used was sodium azide, a compound known for its acute toxicity. The reported chitosan derivatives were synthesized without any requirements of sodium azide, and subsequent cell viability assay provided sufficient evidence of their non-toxicity and safety.

#### Fourier transform infrared (FT-IR) analysis

**Fig 2:**
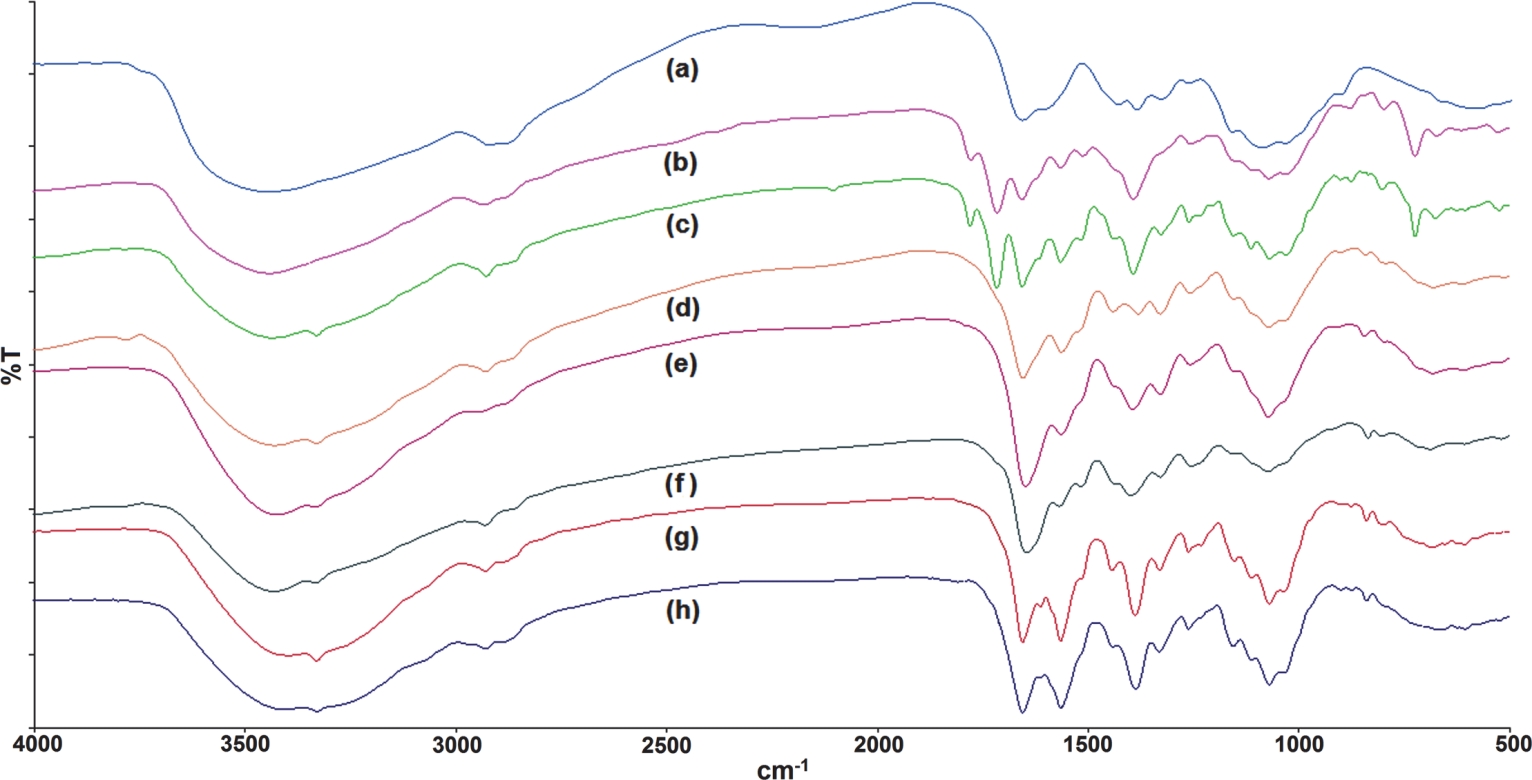
Fourier transform infrared (FTIR) spectra. Figure showing fouriertransforminfrared (FTIR) spectra of (a) Chitosan, (b) *N*-phthaloyl-chitosan, (c) *N*-phthaloyl-chitosan O-prop-2-ynyl carbamate, (d) derivative 1, (e) derivative 2, (f) derivative 3, (g) derivative 4, and (h) derivative 5.

The infrared spectra (Fig 2) for low-molecular-weight (LMW) chitosan **(a)** exhibited the expected characteristic bands as previously reported [25]. A broad and intense band at 3450–3200 cm^-1^ represented overlapping of O-H, N-H symmetric and asymmetric stretching. In addition, the characteristic peaks for C-H stretching, amide I and II, N-H bending of primary amines, and amide III can be seen at 2879, 1652, 1592, and 1325 cm^-1^,respectively. Moreover, the peaks at 1429, 1381, and 1030 cm^-1^ indicate the presence of primary alcohol,-CH_3_ symmetrical angular deformation, and C-O-C stretching vibration in the glucopyranose ring, respectively. Specific bands denoting β-(1,4) glycoside bridge can be seen at 1153 and 898 cm^-1^[36].

The additional peaks at 1780 cm^-1^ and 1713 cm^-1^ (C = O stretching bands) observed in the N-phthaloyl chitosan (**b)**,correspond to the specific phthalimide group [36]. Furthermore, the peaks representing imide C = C stretching, = C-H out of plane deformation, and aromatic ring deformation are present at 1562, 720, and 530 cm^-1^, respectively.

The formation of *N*-phthaloyl-chitosan *O*-prop-2-ynyl carbamate**(c)** was confirmed by peaks at 3319 cm^-1^ (≡C-H stretching vibration), 2124 cm^-1^ (C≡C stretching band), 1567 cm^-1^ (N-H deformation and C-N stretching), and 1259 cm^-1^ (coupled C-N and C-O stretching vibration from carbamate group).

Following azide-alkyne coupling and subsequent depthaloylation (**d,e,f,g,h)**, the reaction of azide compounds, 1-azidoadamantane, azidobenzene solution, 1-azidomethyl-2-methyl benzene solution, azidomethyl phenyl sulphide, and 2-azidomethyl-1-Boc-pyrolidine, respectively with *N*-phthaloyl-chitosan O-prop-2-ynyl carbamate displayed N-C = N bending at 838 cm^-1^ indicating the presence of triazole ring whereas the disappearance of C = O stretching, = C-H out of plane deformation, and aromatic ring deformation at 1700, 720, and 530 cm^-1^ confirmed the removal of pthaloyl moiety.

#### ^1^H Nuclear magnetic resonance(1H NMR) analysis

**Fig 3:**
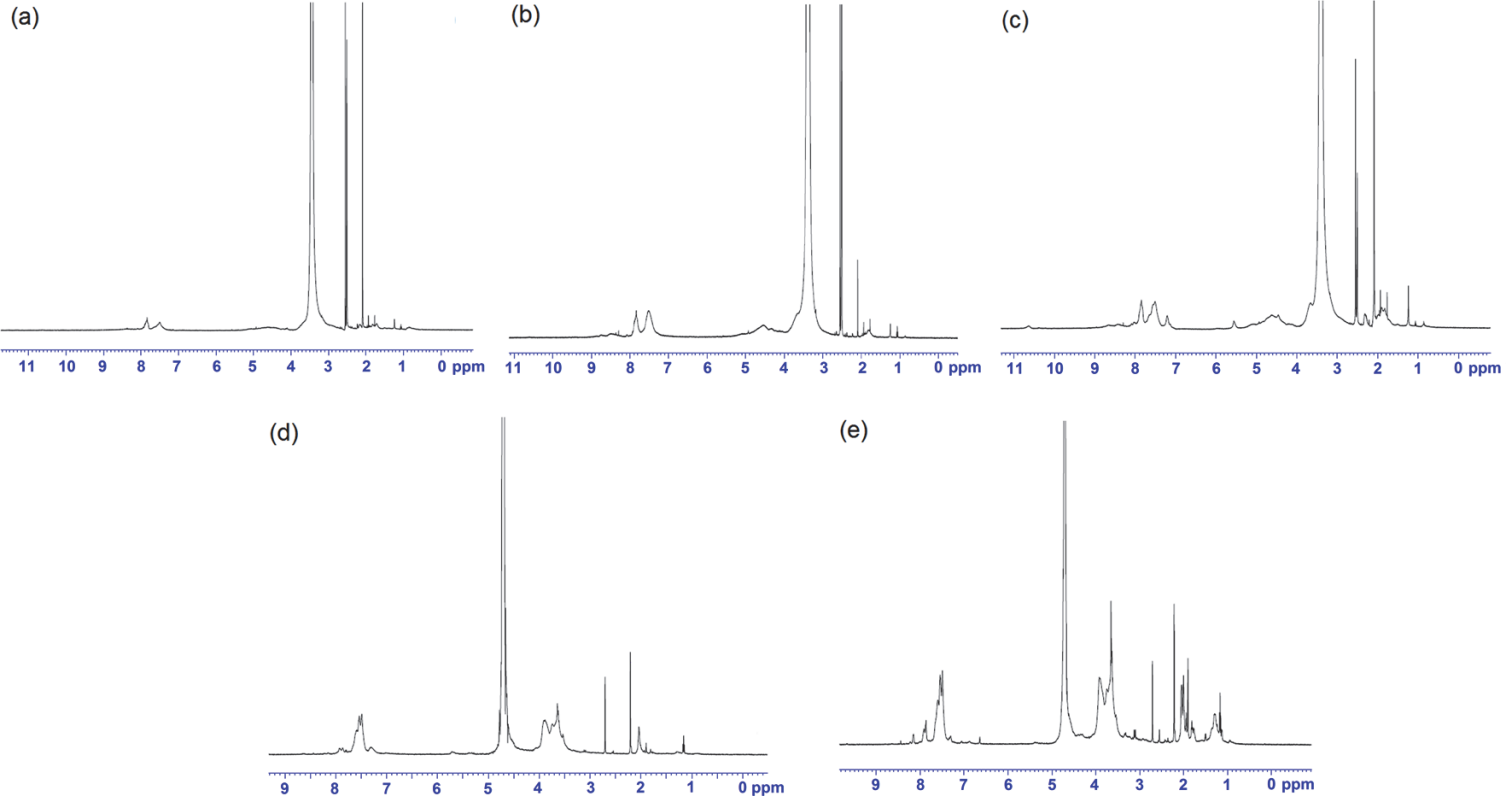
^1^H Nuclear magnetic resonance (1H NMR) spectra. Figure showing ^1^Hnuclear magnetic resonance (^1^HNMR) spectra of (a) derivative 1, (b) derivative 2, (c) derivative 3, (d) derivative 4, and (e) derivative 5.

Derivatives were identified and characterized by obtaining the ^1^H NMR spectra for all final solid fractions, following collection and freeze drying (Fig 3).

*Chitosan O-(adamantane) triazolylcarbamate* **(1)**

δ 7.49 ppm(s, 1H), δ 4.8–4.3 ppm (m, 4H), δ 4.1–3.1 ppm (Pyranose), δ 2.09 ppm (m, 15H).

*Chitosan O-(benzoyl) triazolylcarbamate* **(2)**

δ 7.84–7.83 ppm (m, 5H), δ 7.52 ppm (s, 1H), δ 4.96–4.20 (m, 4H), δ 3.96–3.01 ppm (Pyranose), 2.09 ppm (m, 3H).

*Chitosan O-(1-methylbenzene) triazolylcarbamate* **(3)**

δ 7.84–7.82 ppm (m, 4H), δ 7.50 ppm (s, 1H), δ 4.62 ppm (s, 2H), δ 4.45 ppm (s, 2H), δ 3.92–3.17 ppm (Pyranose), δ 2.09 ppm (m, 3H).

*Chitosan O-(1-methyl phenyl sulphide) triazolylcarbamate* **(4)**

δ 7.9–7.5 ppm (m, 5H), δ 7.49 ppm (s, 1H), δ 4.8–4.7 ppm (m, 6H), δ 4.1–3.4 ppm (Pyranose).

*Chitosan O-((R) (1-methyl)-1-Boc-pyrrolidine) triazolylcarbamate* **(5)**

δ 7.90–7.54 ppm (m, 1H), δ 7.49 ppm (s, 1H), δ 5.30–4.61ppm (m, 6H), δ 3.92–2.54 ppm (pyrrolidine), δ 4.61–3.12 ppm (overlapped pyranose), δ 2.21 ppm (s, 9H, boc).

#### Elemental analysis of chitosan and its derivatives

The composition of carbon, hydrogen, nitrogen, and sulfur elements of chitosan and its derivatives are provided in (Table 1). The substitution of azide-alkyne groups on chitosan occurred at C-6 position.The increase in the percentage of carbon atoms indicates the introduction of azide-alkyne groups. The degree of substitution (DS) was calculated by data obtained from elemental analysis [14,32,37] using the previously reported equation [33].

$$DS\;=\;\left[\left(\frac{C}{N}\right)-\left(\frac{C}{N}\right)˳\right]/n$$

Where, C/N is the ratio of carbon to nitrogen of chitosan derivatives, (C/N)_o_ is the ratio of carbon to nitrogen of the native chitosan, and “*n*” is the number of additional carbon atoms introduced after modification. The DS values obtained were 0.13, 0.23, 0.18, 0.21, and 0.24 for chitosan derivatives1,2,3,4 and 5, respectively.

**Table 1:** Elemental analysis of chitosan and derivatives.

	C	H	N	S
Chitosan	40.04	7.11	7.51	—-
Derivative 1	54.55	6.88	15.76	—-
Derivative 2	50.39	6.02	17.18	—-
Derivative 3	52.72	5.82	16.83	—-
Derivative 4	48.41	5.25	16.09	7.14
Derivative 5	48.98	6.77	17.13	—-

#### Thermogravimetric analysis (TGA)

**Fig 4:**
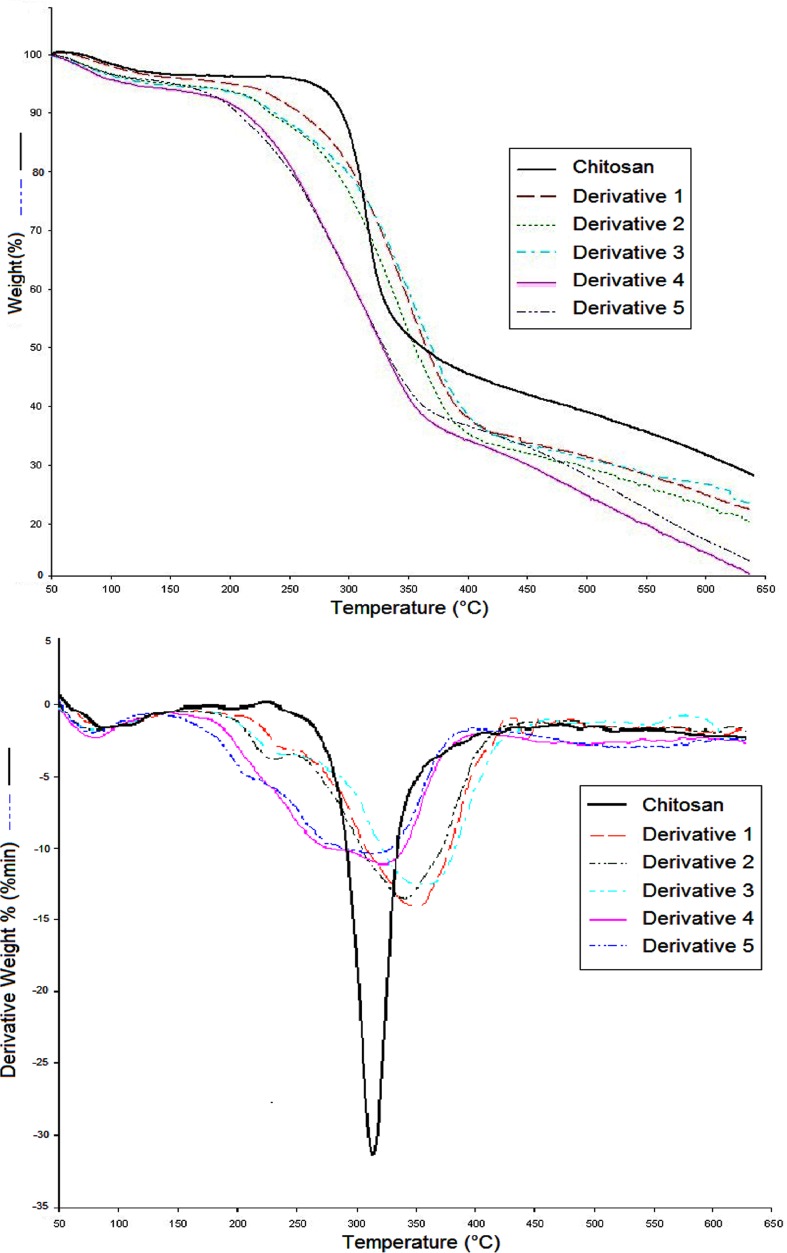
TGA of chitosan and derivaties. Figure showing the thermograms of chitosan and derivativesby using thermogravimetric analysis (TGA).

Thermograms for chitosan and its derivatives (Fig 4) displayed two distinct stages of thermal decomposition. In all the samples, an initial weight loss of 3–6% at approximately 80 °C was observed, and this can be attributed to the loss of adsorbed and bound water. For chitosan, the second stage of decomposition started at 255 °C and reached maximum at 390 °C resulting in a weight loss of 51%. In case of derivatives, decomposition of chitosan backbone chain and cleavage of substituent groups commenced at 210 °C and attained maximum decomposition at 421 °C with weight loss of 62%. In derivatives 4 and 5, the weight further decreased as temperature was raised to 650 °C. This shift in stability and change in decomposition temperature of chitosan derivatives is expected and can be attributed to loss in hydrogen bonding caused by derivatization of the OH group, the introduction of the triazole ring in the chitosan molecule, and the increased hydrophilicity of the derivatives, particularly 4 and 5 [38].

#### Differential scanning calorimetry (DSC)

**Fig 5:**
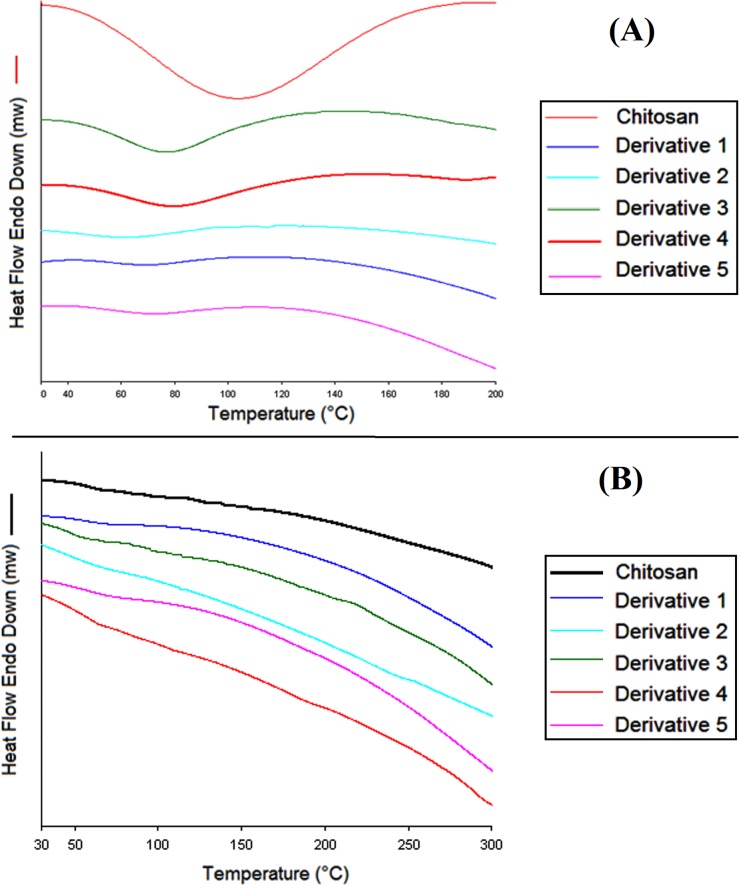
DSC of chitosan and derivatives. (A) First DSC curve of chitosan and derivatives obtained at a heating rate of 10 °C /min and (B) Second DSC curve of dry chitosan and derivatives obtained at a heating rate of 10 °C /min/.

In addition to TGA which is used to evaluate thermal decomposition of the derivatives, DSC was carried out to determine thermal glass transition temperature (Tg). Derivatives were characterized to identify any changes of endothermic phase, following the introduction of azide-alkyne compounds. All the samples were completely freeze dried at -110°C for 48 h prior to DSC analysis. The observed endothermic peaks for chitosan and its derivatives were 115°C and between 65–85°C, respectively (Fig 5). It might be attributed to the adsorbed water due to the hygroscopicity of polymer and its derivatives. A second heating run of DSC, after heatingto100°C, followed by 5 min hold and cooling to 30°C was performed to eliminate the influence of moisture. The scan did not show any specific endothermic transition upto 300°C (Fig 5). This indicated the absence of crystallinity or any phase change during heating process which is in accordance to previous works with chitosan [38,39].Accurate Tg value for chitosan is difficult to be determined due to its hygroscopic property [40,41]. Different Tg values for chitosan have been reported by temperature modulated DSC, e.g. 203°C, 161°C, 150°C, and 118°C in dry state and 61°C in the presence of water. These observations provide evidences that water may form intermolecular hydrogen bonds and acts as plasticizer in chitosan [40,41].

#### Scanning electron microscopy (SEM)

**Fig 6:**
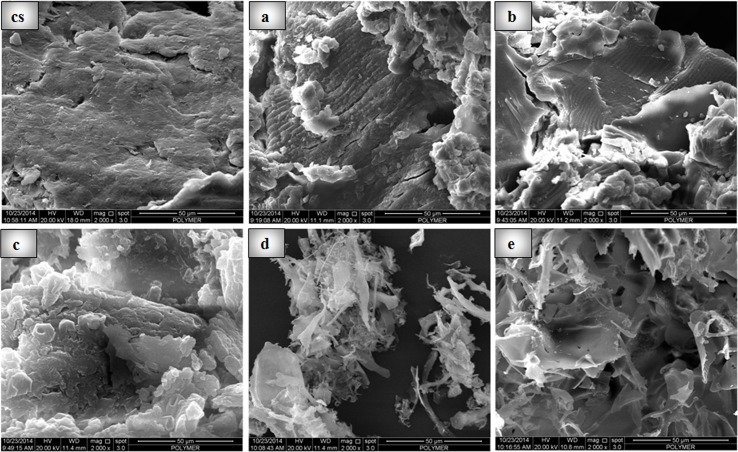
SEM images of chitosan and derivatives. Figure showing scanning electron micrographs (CS) chitosan(a) derivative 1, (b) derivative 2, (c) derivative 3, (d) derivative 4, and (e) derivative 5.

The surface morphology of chitosan and freeze dried powder of chitosan derivatives was analyzed by SEM. All the images were recorded at the same magnification. As shown in (Fig 6), the chitosan exhibited a nonporous, smooth membrane, contain layers of sheets; aggregated, crumpled and closely associated with each other to form a continuous conducting network. The edges appeared crumpled, folded, and closely restacked that resemble with rippled silk waves whereas derivatives are exfoliated into thin large flakes with wavy wrinkles. These images imply that significant structural changes had been occurred, probably due to breaking of hydrogen bonds present in the parent chitosan after the cycloaddition of alkyne-azide conjugates.

#### Particle size, surface charge, and morphology of nanoparticles (NPs)

**Fig 7:**
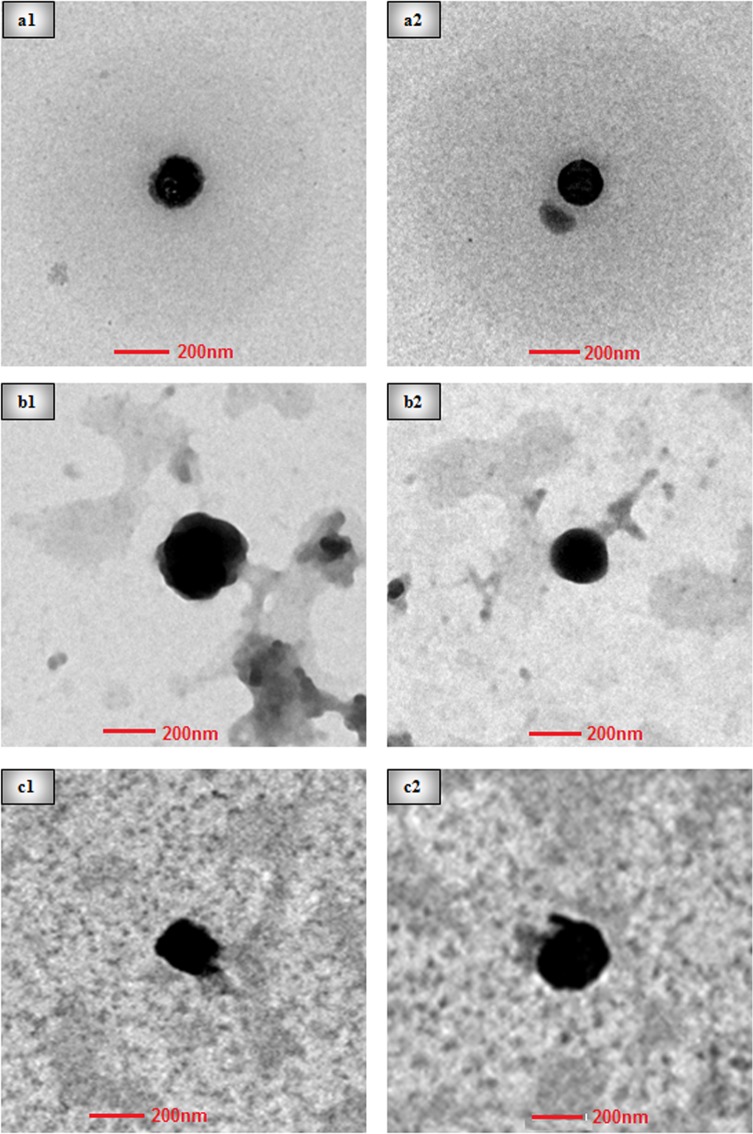
TEM images of chitosan derivatives nanoparticles. Figure showing transmission electron micrographs(a1, a2), (b1, b2), and (c1, c2) represents CSNP3, CSNP4, and CSNP5 respectively.

The NPs of chitosan derivatives 3, 4, and 5 (CSNP3, CSNP4, and CSNP5, respectively) were found to have smaller particle size and narrow range of polydispersity index (PDI), whereas NPs of derivatives 1 and 2 (CSNP1 and CSNP2) exhibited larger particle size and PDI range rendering them unsuitable for further characterization. Small particle size with a narrow range of PDI indicated a uniform distribution in CSNP3, CSNP4, and CSNP5 (Table 2, p<0.05). Since concentrations affect the electrostatic interaction between chitosan derivatives and cross-linker sodium tripolyphosphate (TPP), fabrication of NPs was optimized using a moderate concentration of derivatives (2 mg/mL) and TPP (0.8 mg/mL). Positive zeta potential values of >20 mV indicated the formation of stable nanoparticles with a good surface charge. The morphology of NPs observed by using transmission electron microscopy (TEM) is presented in (Fig 7). NPs of CSNP3 and CSNP4 were more spherical in shape and had a smoother surface in comparison to CSNP5. In general, smaller particles posses greater surface area to volume ratio which provide higher chance of contacting and getting through the cell wall and membrane of bacteria or fungi compared to larger particles or bulk/free form polymers. We have previously reported the enhanced antibacterial and antifungal activities of smaller CSNP relative to the larger ones [27,29]. At nanoscale, the nanoparticles showed a unique optical, electromagnetic and mechanical properties, largely varies from their bulk material. Thus, these properties render the nanoparticles into more effective tool against bacteria and fungi.

**Table 2:** Particle size, polydispersity, zeta potential, antibacterial and antifungal activities of CSNP3, CSNP4 and CSNP5.(n = 3).

CSNP	Particle size	Polydispersity index	Zeta potential	MIC / MBC (μg/mL)						MIC / MFC (μg/mL)		
	(nm)	(μ2/Γ 2)	(mV)	Gram positive bacteria			Gram negative bacteria			Fungi		
				*S*. *aureus*	*B*. *cereus*	*B*. *subtilis*	*E*. *coli*	*A*. *schindleri*	*P*. *aeruginosa*	*A*. *niger*	*C*. *albicans*	*F*. *solani*
**CSNP3**	181.03 ± 12.73	0.49 ± 0.02	22.14 ± 0.89	3.13/3.13	1.56/3.13	1.56/3.13	6.25/6.25	6.25/25	3.13/3.13	375/1500	94/750	375/1500
**CSNP4**	222.04 ± 19.01	0.33 ± 0.04	24.12 ± 1.41	3.13/3.13	3.13/6.25	3.13/6.25	6.25/6.25	6.25/12.5	6.25/12.5	188/375	94/750	375/1500
**CSNP5**	236.50 ± 14.32	0.22 ± 0.02	22.40 ± 6.25	12.5/25	12.5/12.5	12.5/12.5	25.0/50.0	25.0/25.0	12.5/25.0	750/3000	188/1500	750/3000

#### Bacterial morphology

**Fig 8:**
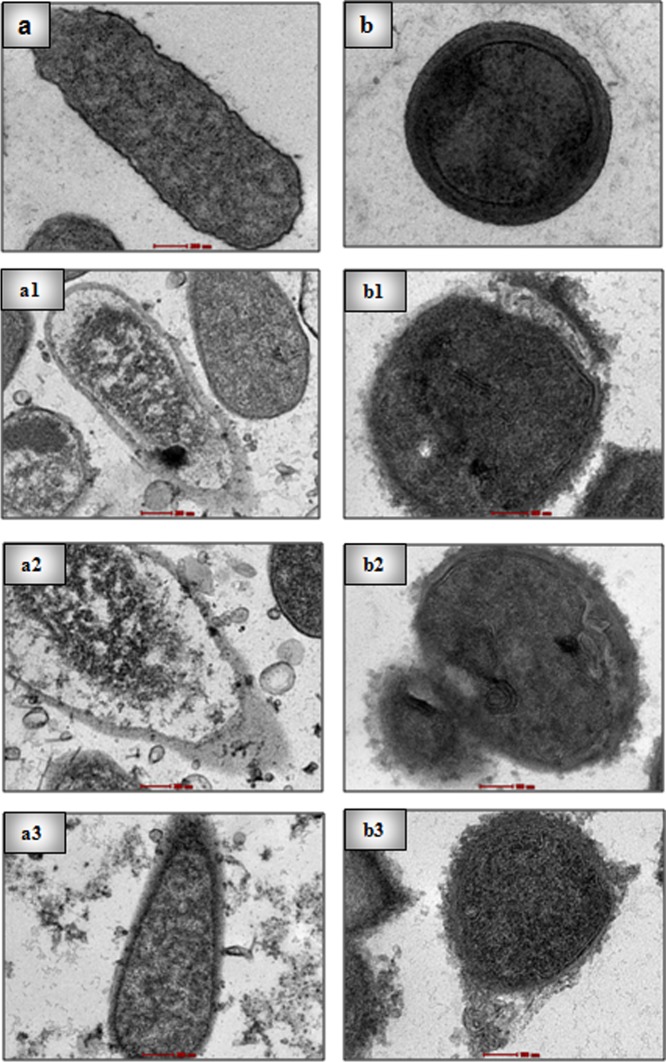
TEM images of bacteria. Figure showing morphology of bacteria. (a) untreated*E*. *coli* (b) untreated *S*. *aureus*. (a1-a3) show*E*.*coli* and (b1-b3) show *S*. *aureus* treated with CSNP3, CSNP4, andCSNP5.

The morphology of *Escherichia coli* and *Staphylococcus aureus* following exposure to NPs was visualized by TEM. The cell wall of untreated bacterium was fairly intact and without any significant rupture. However, treatment with CSNP3, CSNP4, and CSNP5 compromised and disrupted the cell wall of *E*. *coli* and *S*. *aureus* and is shown in (Fig 8). The formation of pores and hollow structures on the cell wall, irregularly shaped cells, and scratched cell surface support the finding that CSNP3, CSNP4, and CSNP5 have the ability to break bacterial envelopes and damage the plasma membrane, leading to the bactericidal effects.

### Evaluation of biological activities

#### Antibacterial activities

MIC is an indicator of antibacterial property; higher MIC correlates to a lower antibacterial activity and vice versa. Negative (untreated) and positive (gentamicin) controls were evaluated simultaneously with the chitosan derivatives and their NPs.

Most of our derivatives and NPs demonstrated an improved antibacterial activity than native LMW chitosan. This is most likely due to the introduction of triazole functionality; previous studies have shown that the antibacterial activity of a series of triazole derivatives is equivalent to and better than that of streptomycin and chloramphenicol, respectively against *E*. *coli*[22].The MIC of all derivatives against the tested gram-positive and gram-negative bacteria (Table 3) ranged from 31.3 μg/mL to 250 μg/mL. Derivatives 3 and 4 exhibited better antibacterial activity than the other derivatives and native chitosan. MIC values of NPs (Table 2) also revealed an enhanced antibacterial activity against all strains, particularly gram-positive bacteria. A wide range of polymers; natural, synthetic and derived from natural resin acids have been previously reported with remarkably improved and prolonged antimicrobial activity against different strains of gram-positive and gram negative-bacteria [33,42].

**Table 3:** Antibacterial and antifungal activity of chitosan and derivatives. (n = 3).

Compounds	MIC / MBC (μg/mL)							MIC / MFC (μg/mL)		
	Gram positive bacteria				Gram negative bacteria			Fungi		
	*S*. *aureus*	*B*. *cereus*	*B*. *subtilis*		*E*. *coli*	*A*. *schindleri*	*P*. *aeruginosa*	*A*. *niger*	*C*. *albicans*	*F*. *solani*
**Derivative 1**	62.5 / 250	250 / 500	125 / 250		125 / 250	250 / 500	125 / 500	1500 /—-	375 / 1500	1500 /—-
**Derivative 2**	62.5 / 125	125 / 250	62.5 / 250		125 / 250	125 / 500	125 / 250	1500 /—-	375 / 1500	750 / 3000
**Derivative 3**	31.3 / 62.5	62.5 / 250	31.3 / 62.5		31.3 / 62.5	125 / 250	31.3 / 125	1500 /—-	188 / 1500	750 / 3000
**Derivative 4**	31.3 / 62.5	62.5 / 250	31.3 / 62.5		31.3 / 62.5	125 / 250	31.3 / 125	750 /—-	188 / 1500	750 / 3000
**Derivative 5**	62.5 / 125	62.5 / 250	62.5 / 250		125 / 250	125 / 250	125 / 500	1500 /—-	375 / 3000	1500 / 3000
**Chitosan**	125 / 250	500 /—-	125 / 500		250 / 500	500 /—-	500 /—-	3000 /—-	3000 /—-	3000 /—-

Various factors could contribute to the improved antibacterial properties of chitosan derivatives and their NPs. Besides the cationic nature of chitosan and protonation-mediated solubilization, the number of amino groups linked at C-2 on chitosan backbone has an important role in the electrostatic interaction and formation of nanoparticles. Hence, a substantial number of amino groups could possibly contribute to an increased antibacterial activity. In addition, our results showed that the derivatives and NPs exhibited higher antibacterial activity against gram-positive bacteria than gram-negative bacteria, and this could be because of differences in cell wall composition and structure of the two bacteria types. The lipopolysaccharides, lipoproteins, and phospholipids in the outer membrane of gram-negative bacteria provide a penetration barrier against macromolecules. The cell wall of gram-positive bacteria, on the other hand, is composed of a thin layer of peptidoglycan, teichoic acid, and has plenty of pores that allow easy entrance to foreign molecules,and eventually leads to disruption of cytoplasmic membrane, loss of cytoplasmic constituents, and ultimately cell death. In addition, the higher negative charge on the cell surface of gram-positive bacteria improves chitosan adsorption and contributes to an improved antibacterial activity in comparison to gram-negative bacteria [43].

#### Antifungal properties

MIC of chitosan NPs and derivatives against tested fungal strains is given in (Tables 2 and 3), respectively. Derivatives were found to have an improved antifungal activity against all the three strains of fungi as comparison to native chitosan. Among the synthesized derivatives, derivatives 3 and 4 and their corresponding nanoparticles CSNP3 and CSNP4 showed the highest antifungal activity against *C*. *albicans* with an MIC of 94 μg/mL. *A*. *niger* appeared to be more resistant than the other tested strains to the antifungal effects of derivatives and NPs. The improved antifungal activity could be attributed to the triazolic conjugation since azole compounds are believed to possess antifungal action [22,23]. Moreover, triazoles possess remarkable stability and are not susceptible to cleavage, making them difficult to oxidize or reduce.

In our study, derivatives of chitosan prepared by click chemistry showed lower MIC values suggesting a stronger antifungal action than native chitosan. However, this antifungal property is also dependent on the species of fungus [44]. Previous studies had shown that unmodified chitosan had a higher MIC value (3000–5000 μg/mL) [45].

In the reported study, fabricated NPs showed lower MIC values against all the selected fungal inocula, suggesting that smooth surface, smaller size, and positive surface charge on NPs render them more effective as antifungal agents.

These results also showed the utility of the click reaction of alkyne and azide to form a triazole in synthesizing chitosan derivatives with enhanced antifungal activity. This finding coincides with those of a previous study [46], the results of which established the antifungal efficacy of the five membered ring systems, including triazolefor treating fungal infections such as candidiasis, aspergillosis, and cryptococcal meningitis.The enhanced activity could be an additive effect due to the inhibition of fungal cytochrome P450 dependent conversion of lanosterol to ergosterol by the triazole moiety as well as the existing antifungal activity of chitosan, induces changes in permeability of the fungal membrane [46].Chitosan derivatives interfere with the fungal membrane and causes leakage of its intracellular components. However, *A*.*niger* was found to be more resistant to chitosan derivatives [29], The resistance observed could be related to the fact that *A*. *niger* has shown an increased chitin synthesis in response to the addition of cell wall disturbing compounds.

#### Effect on cell viability

**Fig 9:**
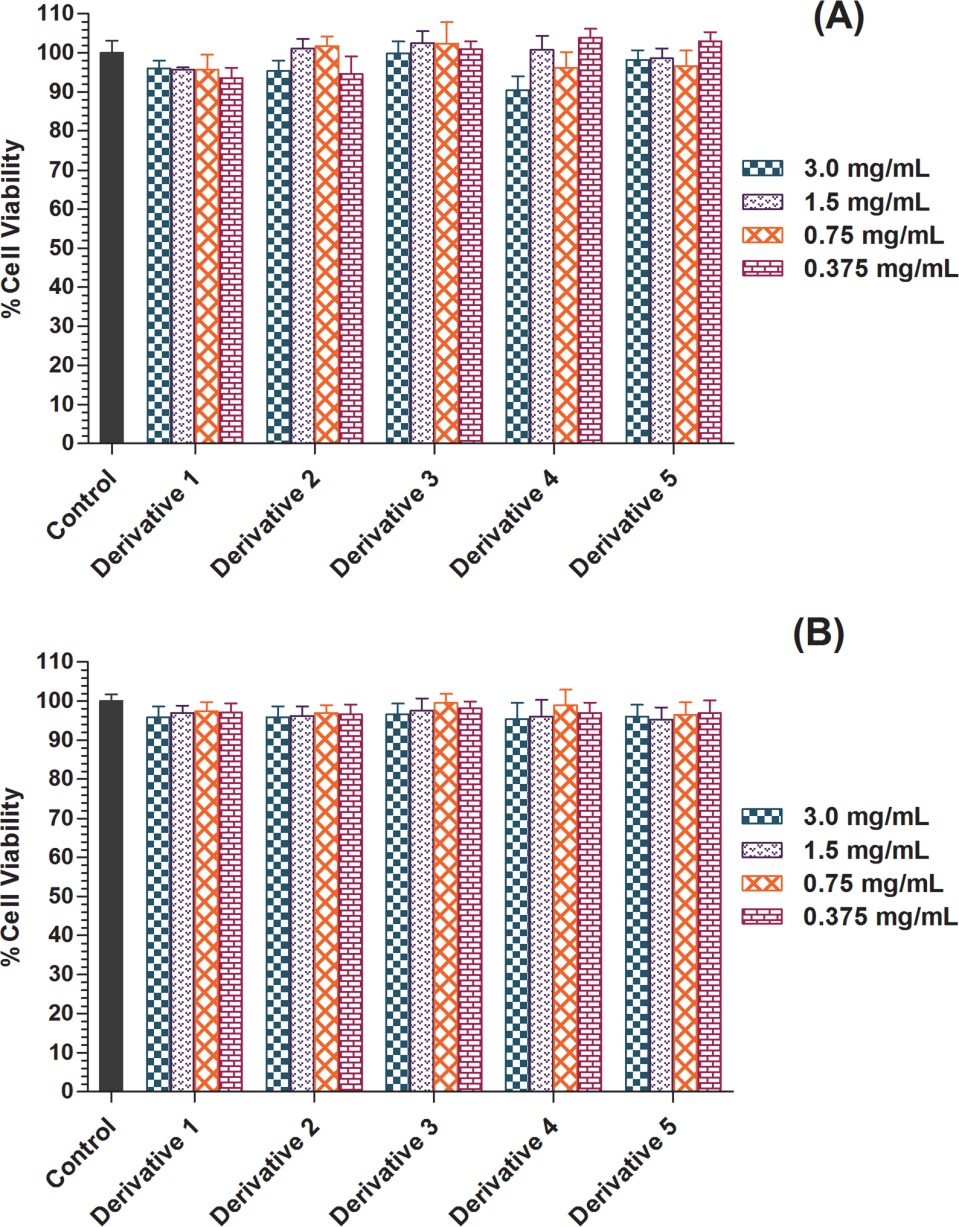
Cell viability of chitosan and derivatives. Figure showing the cell viability effects of chitosan and derivatives on (A) Chinese hamster lung fibroblast cell line V79 and (B) Human hepatic cell line WRL68. Data was presented asmean ± SD (n = 3).

Cytotoxicity of chitosan derivatives was investigated by using AlamarBlue cell viability assay. Derivatives showed no significant cytotoxicity in V79 and WRL68 cell lines after exposure for 24 h. In comparison to untreated cells, cell viability of V79 and WRL68 cell line was more than 92% and 95%, respectively when exposed to chitosan derivatives (p<0.05) (Fig 9). These results demonstrate that all synthesized chitosan derivatives were relatively non-toxic and safe and could be explored for potential biomedical applications.

#### Hemolytic effect on erythrocytes

Haemolytic activity of chitosan derivatives has been investigated by measuring the lysis of10% erythrocytes suspension using spectrophotometric assay. In this study, haemolytic activity was estimated as HC_50_,the concentration of chitosan derivatives which induced 50% release of hemoglobin from erythrocytes relative to the positive control (total lysis of erythrocytes suspension was achieved by adding 1% Triton X-100). In the literature, the cell specificity of antimicrobial compounds towards bacteria *vs*. red blood cells has been described by selectivity index value$(SI\;=\;\frac{{HC}_{50}}{MIC})\;$[47]. Since the HC_50_ and MIC values depend on various factors e.g. the number of cells used in the assay, sample preparation, pH, solvent, concentration and assay medium, therefore, it is difficult to assess the absolute cell specificity of antimicrobial compounds based on SI alone.

Despites all derivatives exhibited high HC_50_values; SI values were rather low for all fungal strains and some bacterial strains. For example, derivative 3 with MIC and HC_50_ value of 31.3 and 8000 μg/mL respectively against *S*.*aureus* gave an outstanding SI value of >250. On the other hand, the same derivative which had MIC value of 1500 μg/mL against *A*.*niger* gave a very low SI value of only~5 μg/mL (Table 4).In this case, the derivatives which weremore potent displayed a better SI.

**Table 4:** Hemolytic activity of chitosan and derivatives. (n = 3).

		SI = HC_50_ / MIC(μg/ml)								
Compounds	HC_50_(μg/ml)	Gram positive bacteria			Gram negative bacteria			Fungi		
		*S*. *aureus*	*B*. *cereus*	*B*. *subtilis*	*E*. *coli*	*A*. *schindleri*	*P*. *aeruginosa*	*A*. *niger*	*C*. *albicans*	*F*. *solani*
**Derivative 1**	4000	128	32	64	64	32	64	5.3	21.3	5.3
**Derivative 2**	8000	128	64	128	64	64	64	5.3	21.3	10.7
**Derivative 3**	8000	255.6	128	255.6	255.6	64	255.6	5.3	42.6	10.7
**Derivative 4**	4000	127.8	64	127.8	127.8	32	127.8	5.3	21.3	5.3
**Derivative 5**	4000	64	64	64	32	32	32	2.7	10.7	2.7
**Chitosan**	16000	128	32	128	64	32	32	5.3	5.3	5.3

## Conclusions

Chitosan is a unique polymer associated with several biological activities and has attracted much attention recently for further development as an antimicrobial agent. The use of a natural product like chitosan for various pharmaceutical applications is highly recommended because of its environmental and ecologic benefits. In this study, a series of chitosan derivatives have been successfully synthesized by cycloaddition of azide-alkyne compounds. The present approach enables us to design highly chemoselective and biologically active chitosan triazolyl derivatives and formulate them into NPs. All derivatized chitosan triazolyl compounds and their corresponding nanoparticles exhibited better antibacterial and antifungal activities while preserving the non-toxicity of the unmodified chitosan. These findings prove that introduction of desired triazole rings by chemoselective modifications of chitosan, retaining the backbone structure of polymer, and maintaining its intrinsic properties could potentially be used as a powerful tool to design promising chitosan derivatives with well-defined and improved biological properties. Further investigations will be carried out to evaluate additional activities of these derivatives and their nanoparticles while designing and synthesizing more potent compounds in parallel.
